# Chemogenetic Manipulation of Amygdala Kappa Opioid Receptor Neurons Modulates Amygdala Neuronal Activity and Neuropathic Pain Behaviors

**DOI:** 10.3390/cells13080705

**Published:** 2024-04-19

**Authors:** Guangchen Ji, Peyton Presto, Takaki Kiritoshi, Yong Chen, Edita Navratilova, Frank Porreca, Volker Neugebauer

**Affiliations:** 1 Department of Pharmacology and Neuroscience, Texas Tech University Health Sciences Center, 3601 4th St., Lubbock, TX 79430, USA; 2 Center of Excellence for Translational Neuroscience and Therapeutics, Texas Tech University Health Sciences Center, Lubbock, TX 79430, USA; 3 Garrison Institute on Aging, Texas Tech University Health Sciences Center, Lubbock, TX 79430, USA; 4 Department of Pharmacology, Arizona Health Sciences Center, University of Arizona, Tucson, AZ 85721, USA

**Keywords:** KOR, DREADD, chemogenetics, amygdala, pain, neuroplasticity

## Abstract

Neuroplasticity in the central nucleus of the amygdala (CeA) plays a key role in the modulation of pain and its aversive component. The dynorphin/kappa opioid receptor (KOR) system in the amygdala is critical for averse-affective behaviors in pain conditions, but its mechanisms are not well understood. Here, we used chemogenetic manipulations of amygdala KOR-expressing neurons to analyze the behavioral consequences in a chronic neuropathic pain model. For the chemogenetic inhibition or activation of KOR neurons in the CeA, a Cre-inducible viral vector encoding Gi-DREADD (hM4Di) or Gq-DREADD (hM3Dq) was injected stereotaxically into the right CeA of transgenic KOR-Cre mice. The chemogenetic inhibition of KOR neurons expressing hM4Di with a selective DREADD actuator (deschloroclozapine, DCZ) in sham control mice significantly decreased inhibitory transmission, resulting in a shift of inhibition/excitation balance to promote excitation and induced pain behaviors. The chemogenetic activation of KOR neurons expressing hM3Dq with DCZ in neuropathic mice significantly increased inhibitory transmission, decreased excitability, and decreased neuropathic pain behaviors. These data suggest that amygdala KOR neurons modulate pain behaviors by exerting an inhibitory tone on downstream CeA neurons. Therefore, activation of these interneurons or blockade of inhibitory KOR signaling in these neurons could restore control of amygdala output and mitigate pain.

## 1. Introduction

Chronic pain affects an estimated 20% of the worldwide population annually [1]. This multifaceted condition includes sensory, emotional-affective, and cognitive dimensions that pose considerable hurdles to the development of effective and well-tolerated treatments, as current therapies are often met with limited success and can lead to undesirable side effects [2]. An incomplete understanding of underlying chronic pain mechanisms may hinder the identification of effective therapeutic targets. While maladaptive neuroplasticity has been associated with the persistence of chronic pain [3], there remains an urgent need to investigate underlying cellular and synaptic mechanisms to develop novel treatment strategies.

In this context, the amygdala has emerged as a limbic brain structure that is critically involved in the emotional-affective component of pain and pain modulation [4,5,6] by assigning emotional value to pain-related sensory information [7,8]. The central nucleus of the amygdala (CeA) serves major amygdala output functions [5,9] and is implicated in behavioral, emotional, and cognitive responses to pain states [10,11,12,13]. The CeA receives nociceptive information directly from the parabrachial nucleus (PB) in the brainstem as well as multimodal sensory information from thalamocortical regions that project to the lateral–basolateral amygdala (BLA) circuitry [5,7]. Changes in amygdala neuroplasticity have been linked to pain-related behaviors in multiple pain conditions [5,14,15]. A greater understanding of the specific mechanisms that govern amygdala neuroplasticity in pain states could provide further insight into therapeutic development.

One area of potential interest lies in the role of opioids and opioid receptor functions within the amygdala. Although the mu-opioid receptor (MOR) predominately mediates analgesic effects, the kappa opioid receptor (KOR) has been linked to aversiveness, anxiety, and stress responses via limbic brain regions that include the amygdala [10,16,17,18,19]. The amygdala has high expression of the KOR, as demonstrated by earlier radioligand binding, immunohistochemical, and in situ hybridization experiments [10,20,21]. Dynorphin, an endogenous agonist for the KOR, has been implicated in aversive behavioral responses to stressors [22] and has been found to be synthesized, along with its precursor prodynorphin, by many neurons in the lateral CeA (CeL) [23,24]. Our previous studies have implicated KOR function in averse-affective pain-like behaviors that were elicited by the inhibition of synaptic inhibition (i.e., disinhibition) at the PB-CeA synapse following the administration of a KOR agonist in naïve rats [25]. We have also shown diminished pain-related behaviors after blockade with a KOR antagonist that corresponded to increased synaptic inhibition in neuropathic [26] and functional [27] pain models. However, the effects of KOR-expressing CeA neurons on downstream neurons and behaviors following direct CeA-KOR activation or inactivation remain to be determined.

The purpose of this study was to examine the specific functions of the CeA-KOR system in modulating neuropathic pain-related amygdala mechanisms and behaviors via a targeted chemogenetic approach using Designer Receptors Exclusively Activated by Designer Drugs (DREADDs) [28]. We delineated the effects of precise CeA-KOR activation or inhibition in a mouse model of neuropathic pain on pain-related mechanical sensitivity, emotional-affective responses, and anxiety-like behaviors. Furthermore, we explored the consequences of chemogenetic CeA-KOR activation or inhibition on downstream CeA neuron excitability and synaptic transmission at the PB-CeA synapse. Our results reveal a pro-nociceptive role of KOR system activation (activation of Gi-signaling in KOR neurons) within the CeA and highlight its potential as a target for novel neuropathic pain therapies. This study not only clarifies the functional dynamics of the CeA-KOR system in pain modulation but also provides support for the use of innovative therapeutic tools in neuropathic pain management.

## 2. Materials and Methods

### 2.1. Animals

Male transgenic KOR-Cre mice (Jax Labs: Oprk1tm1.1(cre)Sros/J; Strain #: 035045) were housed in a temperature-controlled room under a 12 h light/dark cycle with unrestricted access to food and water. On each experimental day, mice were transferred from the animal facility and allowed to acclimate to the laboratory for at least 1 h prior to testing. All experimental procedures were approved by the Institutional Animal Care and Use Committee (IACUC) at Texas Tech University Health Sciences Center (TTUHSC) and conformed to the guidelines of the International Association for the Study of Pain (IASP) and the National Institutes of Health (NIH).

### 2.2. Neuropathic Pain Model

The well-established spinal nerve ligation (SNL) model of neuropathic pain [29] was used to generate stable and long-lasting peripheral neuropathy. Mice were anesthetized with isoflurane (3–4% induction, 2% maintenance) and underwent surgery in which the left L5 spinal nerve was tightly ligated using 6-0 sterile silk. In the sham-operated control group, the nerve was exposed but not ligated.

### 2.3. Chemogenetic Manipulations

For the chemogenetic manipulations of KOR neurons in the CeA, a Cre-inducible viral vector encoding Gi-DREADD (AAV5-hSyn-DIO-hM4D(Gi)-mCherry, 0.3–0.5 μL, titer ≥ 7 × 10^12^ vg/mL, Addgene (Watertown, MA, USA, Catalog #: 44362-AAV5) or Gq-DREADD (AAV5-hSyn-DIO-hM3D(Gq)-mCherry, 0.3–0.5 μL, titer ≥ 7 × 10^12^ vg/mL, Addgene, Catalog #: 44361-AAV5) was injected stereotaxically into the right CeA of heterozygous KOR-Cre mice for specific chemogenetic inhibition or activation of KOR neurons in the CeA. A selective DREADD actuator (deschloroclozapine, DCZ) was used for behavioral (100 μg/kg DCZ in saline, i.p., administered for 7 days) or electrophysiological brain slice (0.5 μM DCZ in artificial cerebrospinal fluid, ACSF) experiments. Appropriate controls were used, such as Cre-dependent mCherry-expression (Dio-mCherry), vehicle (saline, i.p.), or ACSF (brain slice).

### 2.4. Experimental Protocol

Behavioral or electrophysiological studies of the chemogenetic inhibition or activation of KOR neurons (see Section “Chemogenetic manipulations”) in the CeA were conducted in sham and neuropathic KOR-Cre mice (see Section “Neuropathic pain model”). The Cre-inducible viral vector encoding hM4Di or hM3Dq was injected stereotaxically into the right CeA 4–5 weeks before behavioral or electrophysiological testing. In behavioral experiments, a selective DREADD actuator (DCZ) was given systemically (100 μg/kg in saline, i.p., administered for 7 days). Sensory and affective behaviors (mechanosensitivity, vocalizations, light/dark box test) were measured 30 min after the final day injection. In brain slice physiology experiments, the effects of DCZ (0.5 μM) on KOR neurons (mCherry positive neurons) and non-KOR neurons in the CeL were measured (see “Brain Slice Electrophysiology”). To determine the effects of direct chemogenetic activation or inhibition of KOR-positive neurons, resting membrane potentials and excitability (action potential firing) were recorded before and after DCZ application. For outcome measures of downstream effects on non-KOR neurons in the CeL, neuronal excitability (action potential firing) and electrically evoked synaptic activity from the PB were measured before and after the DCZ application. In all experiments, neuronal recordings were performed in the right CeA because of prior evidence for lateralization of KOR signaling and pain processing to the right CeA [30,31,32,33,34].

### 2.5. Pain-Related Behaviors

*Mechanosensitivity.* Mechanical withdrawal thresholds were measured using a plantar electronic von Frey anesthesiometer (IITC Life Science, Woodland Hills, CA, USA), where the tip was applied perpendicularly to the base of the third or fourth toe of the hind paw with increasing force until a flexion reflex was elicited. This force was automatically recorded as the paw withdrawal threshold (in grams). The average of triplicate measurements taken at least 30 s apart was used [35].

*Vocalizations.* Ultrasonic vocalizations were used to assess the emotional-affective responses to noxious stimuli in different experimental pain conditions [36,37,38,39,40]. Ultrasonic (25 ± 4 kHz) vocalizations were measured using a bat detector that was connected to a filter and amplifier (UltraVox four-channel system; Noldus Information Technology, Leesburg, VA, USA) as described in our previous work [41]. Animals were briefly anesthetized with isoflurane (2% precision vaporizer) to allow for gentle and slightly restrained placement in a custom-designed recording chamber that permitted hindlimb access for mechanical test stimuli. After habituation to the chamber, brief (15 s) innocuous (100 g/6 mm^2^) or noxious (300 g/6 mm^2^) mechanical stimuli were applied to the hind paw as described before [41], using a calibrated forceps with a force transducer to monitor the applied force (in grams). The duration of ultrasonic vocalizations was analyzed in each mouse for 1 min after the onset of the mechanical stimulus using Ultravox 2.0 software (Noldus Information Technology, Leesburg, VA, USA).

*Anxiety-like behavior.* The light/dark box (LDB; Noldus Information Technology) test [42] was used to measure anxiety-like behavior in mice. An acrylic LDB was divided into one dark box (17 cm × 40 cm × 40 cm) with black walls and a black lid cover and one lightbox (25 cm × 40 cm × 40 cm) with transparent walls and a transparent lid cover. A door (5 cm × 5 cm) between the two boxes allowed mice to have free access to both boxes. The mouse was first placed in the light box, and the exploratory behavior was recorded for 15 min using a computerized videotracking and analysis system (EthoVisionXT 11 software, Noldus Information Technology). The duration in each chamber was calculated for the first 5 min. Avoidance of the light chamber in the LDB suggests anxiety-like behavior.

### 2.6. Brain Slice Electrophysiology

Whole-cell patch–clamp electrophysiological experiments were performed as described in our previous studies [43,44]. Mice were decapitated, and the brains were rapidly removed and immersed in oxygenated ice-cold sucrose-based slicing solution containing the following (in mM): 87 NaCl, 75 sucrose, 25 glucose, 5 KCl, 21 MgCl_2_, 0.5 CaCl_2_, and 1.25 NaH_2_PO_4_. Coronal slices (400 μm) containing the right CeA were obtained using a Vibratome (VT1200S, Leica Biosystems, Nussloch, Germany) and incubated in oxygenated ACSF at room temperature (21 °C) for at least 1 h before patch recordings. Recording ACSF contained the following (in mM): 117 NaCl, 4.7 KCl, 1.2 NaH_2_PO_4_, 2.5 CaCl_2_, 1.2 MgCl_2_, 25 NaHCO_3_, and 11 glucose. For whole-cell patch recording, a brain slice containing the CeA was transferred to the recording chamber and superfused with oxygenated ACSF (31 °C) at 2 mL/min. Whole-cell patch–clamp recordings were obtained from visually identified mCherry-positive (see “Chemogenetic manipulations”) KOR neurons in the CeA or non-KOR neurons in the CeL using fluorescence microscopy as described previously [25,27]. Borosilicate glass recording electrodes (4–8 MΩ tip resistance) were filled with an intracellular solution containing (in mM): 122 K-gluconate, 5 NaCl, 0.3 CaCl_2_, 2 MgCl_2_, 1 EGTA, 10 HEPES, 5 Na_2_-ATP, and 0.4 Na_3_-GTP; pH was adjusted to 7.2–7.3 and osmolarity to 280 mOsm/kg, and 0.2% biocytin. The K-gluconate internal was chosen as a more physiological solution to allow the analysis of membrane properties, action potential firing, and excitatory and inhibitory transmission in the same cell as in our previous studies [25,27]. Action potentials were evoked by depolarizing current steps (0.5 s) of increasing amplitude (25 pA) from the resting membrane potential in the current clamp. Excitatory and inhibitory postsynaptic currents (EPSCs and IPSCs, respectively) were recorded in voltage–clamp at −70 and 0 mV, respectively, as described previously [25,27].

### 2.7. Histological Verification of Injection and Recording Sites

DREADD-Gi or DREADD-Gq expression in the amygdala was visualized during patch recordings under fluorescent microscopy. To confirm the location of recorded neurons, the recorded slices were fixed in 4% paraformaldehyde in 0.1 M phosphate buffer for 12–24 h at 4 °C. Immunohistochemistry was performed after fixation as described previously [25,45]. Slices were incubated in fluorescently conjugated streptavidin (1:500, Streptavidin, Alexa Fluor 594 conjugate, Life Technologies, Carlsbad, CA, USA) for 12–24 h at 4 °C. The slices were mounted with Vectashield mounting medium with DAPI (Vector Laboratories) and imaged under a confocal microscope (FV3000, Olympus, Center Valley, PA, USA).

### 2.8. Statistical Analysis

All averaged values are presented as the mean ± SEM. Statistical significance was accepted at the level *p* < 0.05. GraphPad Prism 9.0 software was used for all statistical analyses, which were performed on raw data. *t*-tests were used for comparison of two datasets of data with Gaussian distribution and similar variance. For multiple comparisons, a one-way or two-way ANOVA (repeated measures where appropriate) was used with Bonferroni post hoc tests.

## 3. Results

The goal of this study was to investigate the direct chemogenetic manipulation of KOR neurons in the CeA and the impact on downstream CeA neuronal activity and chronic neuropathic pain-related behaviors (4 weeks after induction of SNL) using KOR-Cre mice. Behavioral assays were used to assess the effects of chemogenetic inhibition or activation of CeA-KOR neurons on neuropathic pain behaviors. Brain slice electrophysiology was used to verify the chemogenetic manipulation strategy in CeA-KOR neurons and to assess the effects on downstream non-KOR neurons in the CeL.

### 3.1. Chronic Neuropathic Pain (SNL Model) Behaviors of KOR-Cre Mice

**Figure 1:**
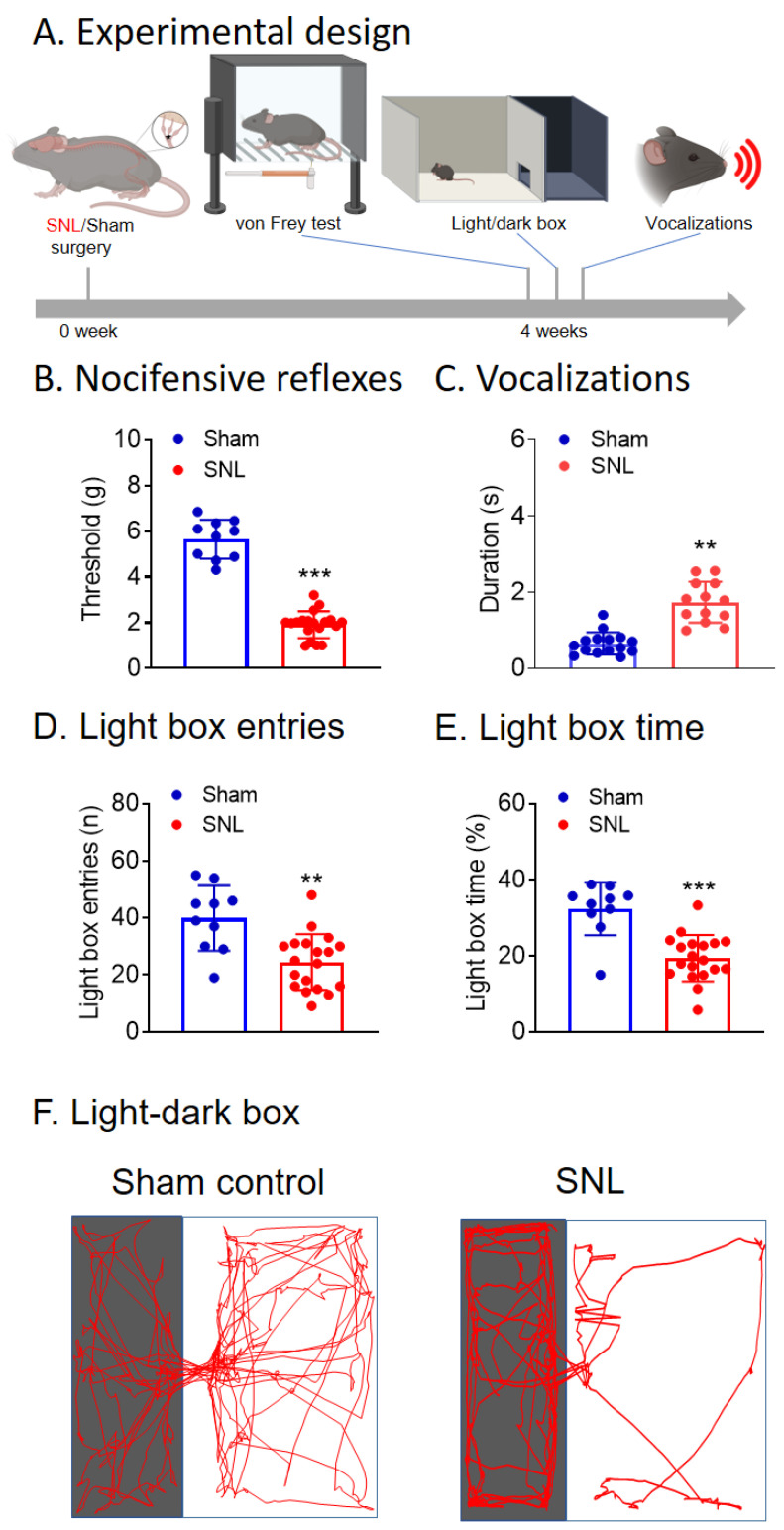
Nocifensive, emotional, and anxiety-like pain behaviors in neuropathic (SNL) KOR-Cre mice. (**A**) Experimental design. (**B**) Mechanical withdrawal thresholds significantly decreased in SNL mice (n = 19, *** *p* < 0.001, unpaired *t*-tests) compared to sham controls (n = 10). (**C**) Duration of ultrasonic vocalizations significantly increased in SNL mice (n = 13, ** *p* < 0.01, unpaired *t*-tests) compared to sham controls (n = 15). In the light/dark box test, SNL mice showed decreased frequency of entries (**D**, n = 19, ** *p* < 0.01, unpaired *t*-tests) and decreased time (**E**, n = 19, *** *p* < 0.001, unpaired *t*-tests) in the light box compared to sham controls (n = 10). (**F**) Representative traces of the light/dark box explorations by a sham and an SNL mouse. Bar histograms show the mean ± SEM. Figure created with BioRender.com.

Chronic neuropathic pain behaviors were measured in KOR-Cre mice 4 weeks after SNL or sham control surgery (Figure 1A). Nocifensive reflexes (Figure 1B) were measured using a plantar electronic von Frey anesthesiometer (see “Pain-related behaviors: Mechanosensitivity”). Mechanical thresholds in SNL mice were significantly decreased compared to sham controls (Figure 1B, *p* < 0.001, unpaired *t*-test, n = 10 sham, n = 19 SNL), confirming that SNL surgery produced mechanical hypersensitivity in KOR-Cre mice. Vocalizations (Figure 1C) in the ultrasonic range were evoked by brief (15 s) noxious mechanical stimulation of the left hindpaw (see “Pain-related behaviors: Vocalizations”). SNL mice showed more ultrasonic vocalizations than sham controls (Figure 1C, *p* < 0.01, unpaired *t*-tests, n = 15 sham, n = 13 SNL), indicating increased emotional-affective pain-related behaviors in SNL mice. Anxiety-like behaviors (Figure 1D,E) were measured using the LDB (see “Pain-related behaviors: Anxiety-like behavior”). SNL mice showed significantly decreased frequency (Figure 1D) and duration (Figure 1E) of entries into the light box of the LDB (*p* < 0.01, *p* < 0.001, unpaired *t*-tests, n = 10 sham, n = 19 SNL). Representative traces of the LDB activity in sham control and SNL KOR-Cre mice are shown in Figure 1F. The data suggest that SNL mice show increased neuropathic pain-related anxiety-like behaviors.

### 3.2. Chemogenetic Inhibition of CeA-KOR Neurons Induces Pain-Like Behaviors in Naïve Mice

**Figure 2:**
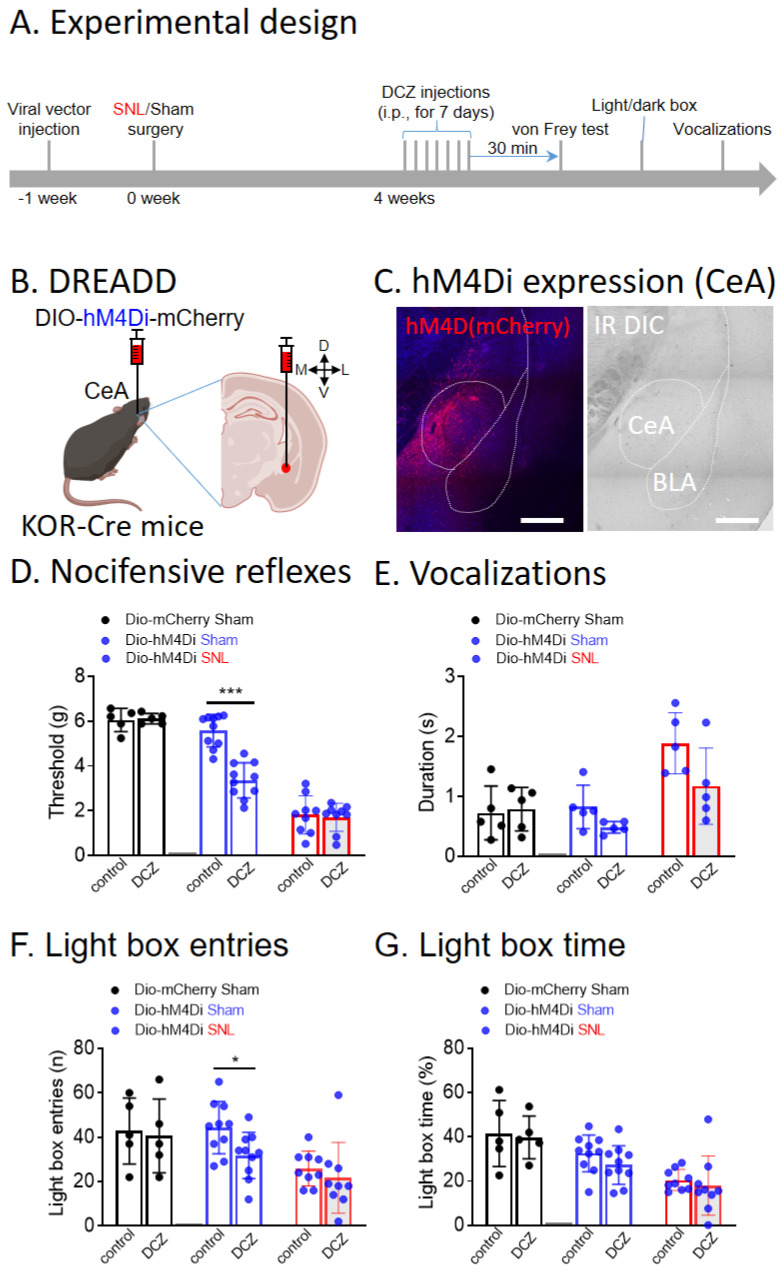
Chemogenetic inhibition of CeA-KOR neurons induces pain-like behaviors. (**A**) Experimental design. (**B**) Diagram of a coronal brain slice (2.30 mm caudal to bregma) shows stereotaxic injection of a Cre-inducible viral vector encoding Gi-DREADD (hM4Di) or control vector (Dio-mCherry) into the right CeA of KOR-Cre mice. (**C**) Confocal image (20× objective, scale bar 200 µm) of KOR neurons expressing hM4Di (mCherry fluorescence) in the CeA. (**D**–**G**) In sham control mice (n = 10), systemic DCZ (100 μg/kg, i.p.; see 2.3 “Chemogenetic manipulations”) significantly decreased mechanical withdrawal thresholds (**D**, n = 10, *** *p* < 0.001, paired *t*-test) and decreased light box entries (**F**, n = 10, * *p* < 0.05, paired *t*-test), but had no significant effect on vocalizations (**E**, n = 5) or light box duration (**G**, n = 10) compared to pre-drug baseline. Systemic DCZ did not have any significant effects in sham mice treated with the control vector (Dio-mCherry, **D**–**G**, n = 5). In SNL mice, systemic DCZ had no significant effects on pain behaviors (**D**–**G**, n = 9). DCZ significantly decreased mechanical thresholds and light box frequency in sham mice, suggesting that chemogenetic inhibition of CeA-KOR neurons induces pain-like behaviors. Bar histograms show the mean ± SEM. Figure created with BioRender.com.

We first tested the effects of chemogenetic inhibition of CeA-KOR neurons on pain behaviors in sham and SNL KOR-Cre mice (Figure 2A). A Cre-inducible viral vector encoding Gi-DREADD (hM4Di) was injected stereotaxically into the right CeA of sham and SNL KOR-Cre mice (Figure 2B), which resulted in the expression of hM4Di (mCherry fluorescence) in CeA neurons (Figure 2C). In sham control mice (n = 10), systemic DCZ (100 μg/kg, i.p.; see “Chemogenetic manipulations”) significantly decreased mechanical withdrawal thresholds (Figure 2D, *p* < 0.001, paired *t*-test) and decreased light box entries (Figure 2F, *p* < 0.05, paired *t*-test), but had no significant effect on vocalizations (Figure 2E, n = 5, paired *t*-test) or light box duration (Figure 2G, n = 5, paired *t*-test) compared to pre-drug baseline. Systemic DCZ did not have any significant effects in sham mice treated with the control vector (Dio-mCherry, Figure 2D–G, n = 5, paired *t*-test). In SNL mice, systemic DCZ had no significant effects on pain behaviors (Figure 2D–G, n = 9, paired *t*-test). The data suggest that inhibition of CeA-KOR neurons can generate nocifensive and anxiogenic behaviors under normal conditions but not in neuropathic pain.

### 3.3. Chemogenetic Activation of CeA-KOR Neurons Decreases Neuropathic Pain Behaviors

**Figure 3:**
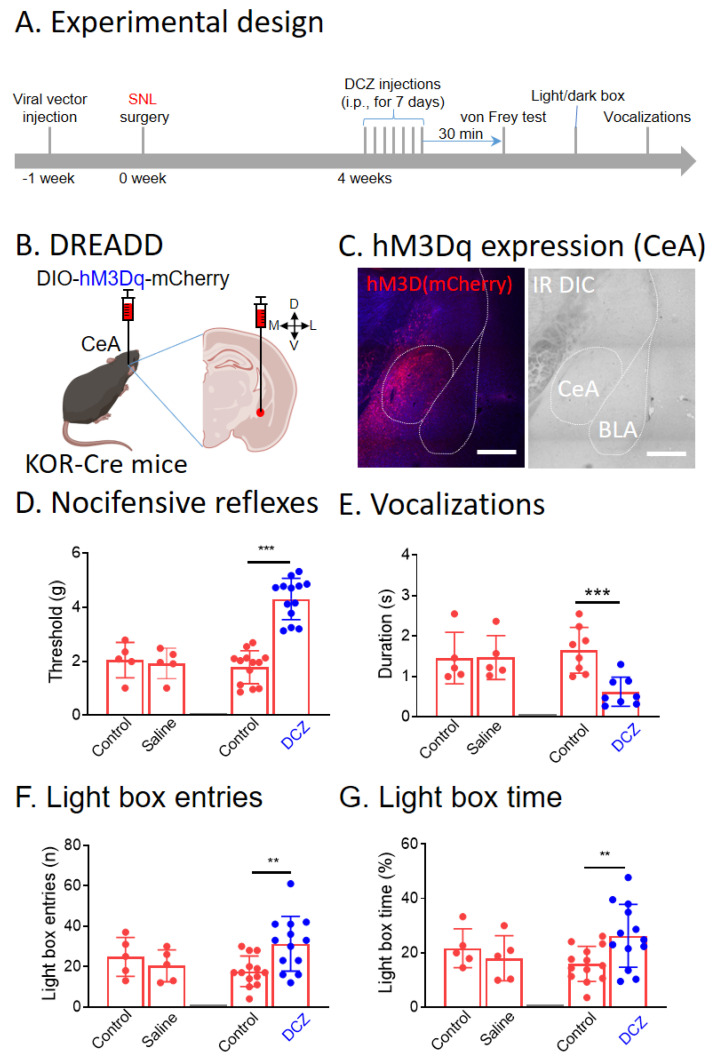
Chemogenetic activation of CeA-KOR neurons reduces neuropathic pain behaviors. (**A**) Experimental design. (**B**) Diagram of a coronal brain slice (2.30 mm caudal to bregma) shows stereotaxic injection of a Cre-inducible viral vector encoding Gq-DREADD (hM3Dq) into the right CeA of SNL KOR-Cre mice. (**C**) Confocal image (20× objective, bar 200 µm) of KOR neurons expressing hM3Dq (mCherry fluorescence) in the CeA. (**D**–**G**). Systemic DCZ (100 μg/kg, i.p.; see 2.3 “Chemogenetic manipulations”) significantly increased mechanical withdrawal thresholds ((**D**), *** *p* < 0.001, n = 13), decreased ultrasonic vocalizations (**E**, n = 8, *** *p* < 0.001, paired *t*-test), and increased light box frequency (**F**, n = 13, ** *p* < 0.01, paired *t*-test) and light box time (**G**, n = 13, ** *p* < 0.01, paired *t*-test). Systemic saline had no effects on mechanical withdrawal thresholds (**D**), ultrasonic vocalizations (**E**), and light box test (**F**,**G**) compared to pre-drug baseline (n = 5). DCZ significantly increased mechanical withdrawal thresholds, decreased ultrasonic vocalizations, and increased light box frequency and time, suggesting that chemogenetic activation of CeA-KOR neurons decreased pain-like behaviors in SNL mice. Bar histograms show the mean ± SEM. Figure created with BioRender.com.

Next, we tested the effects of chemogenetic activation of CeA-KOR neurons on pain behaviors in SNL KOR-Cre mice (Figure 3A). A Cre-inducible viral vector encoding Gq-DREADD (hM3Dq) was injected stereotaxically into the right CeA of SNL transgenic KOR-Cre mice (Figure 3B) to express hM3Dq (mCherry fluorescence) in CeA neurons (Figure 3C). Systemic DCZ (100 μg/kg, i.p.; see 2.3 “Chemogenetic manipulations”) significantly increased mechanical withdrawal thresholds (Figure 3D, *p* < 0.001, n = 13, paired *t*-test), decreased ultrasonic vocalizations (Figure 3E, *p* < 0.001, n = 8, paired *t*-test), and increased light box frequency (Figure 3F, *p* < 0.01, paired *t*-test) and light box time (Figure 3G, *p* < 0.01, n = 13, paired *t*-test) compared to pre-drug baseline. Systemic saline had no significant effects on mechanical withdrawal thresholds (Figure 3D, n = 5, paired *t*-test), ultrasonic vocalizations (Figure 3E, n = 5, paired *t*-test), and light box entries or duration (Figure 3F,G, n = 5, paired *t*-test) compared to pre-drug baseline. These data suggest that activation of CeA-KOR neurons can inhibit nocifensive, emotional, and anxiety-like behaviors in neuropathic pain.

### 3.4. Validation of Chemogenetic Manipulations of CeA-KOR Neurons

**Figure 4:**
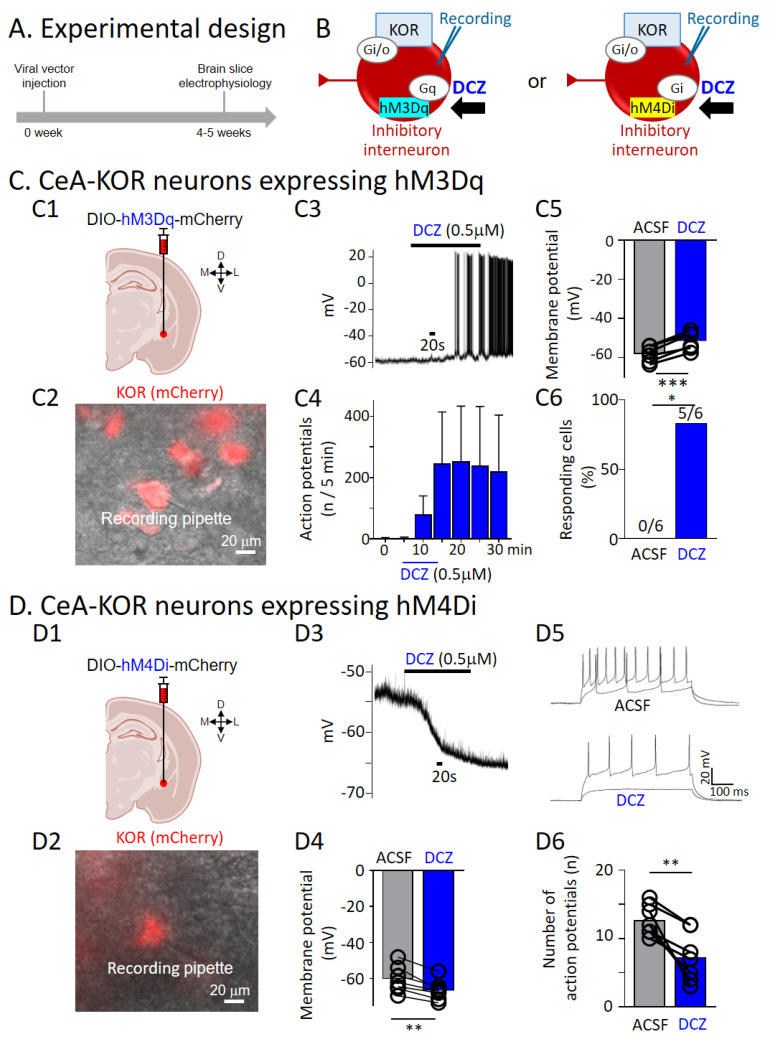
Validation of chemogenetic activation or inhibition of CeA-KOR neurons with electrophysiology. (**A**) Experimental design. (**B**) Whole-cell patch recording from mCherry-expressing KOR neurons following chemogenetic activation (hM3Dq, left) or inhibition (hM4Di, right) (DCZ). (**C**) Chemogenetic activation of KOR neurons in the CeA (CeM and CeL). (**C1**) Coronal brain slice shows stereotaxic injection of a Cre-inducible viral vector encoding Gq-DREADD (hM3Dq) into the right CeA of transgenic KOR-Cre mice. (**C2**) Patch–clamp recording of a CeA-KOR neuron expressing hM3Dq (mCherry). (**C3**) Representative trace of DCZ-induced membrane depolarization and action potential firing in an hM3Dq-expressing CeA-KOR neuron. (**C4**) Averaged number of action potentials in 5 min before, during, and after DCZ (0.5 µM, 15 min, n = 6 neurons, *p* > 0.05, one-way ANOVA). (**C5**) Averaged resting membrane potentials during ACSF pre-drug control and DCZ (0.5 µM, 15 min) (n = 6 neurons, *** *p* < 0.001, paired *t*-test). (**C6**) Percentage of CeA-KOR neurons expressing hM3Dq (mCherry) that responded to DCZ (n = 6 neurons, * *p* < 0.05, Chi-square test). (**D**) Chemogenetic inhibition of KOR neurons in the CeA. (**D1**) Coronal brain slice shows stereotaxic injection of a Cre-inducible viral vector encoding Gi-DREADD (hM4Di) into the right CeA of transgenic KOR-Cre mice. (**D2**) Patch–clamp recording of a CeA-KOR neuron expressing hM4Di (mCherry). (**D3**) Individual trace of DCZ-induced membrane hyperpolarization in an hM4Di-expressing CeA-KOR neuron. (**D4**) Averaged resting membrane potentials during ACSF pre-drug control and DCZ (n = 7 neurons, ** *p* < 0.01, paired *t*-test). (**D5**) Individual traces of the effect of DCZ on the excitability of an hM4Di-expressing CeA-KOR neuron measured with depolarizing current injections from a holding potential of −60 mV. (**D6**) Summary data for action potential firing (n = 7 neurons, ** *p* < 0.01, paired *t*-test). Bar histograms show the mean ± SEM. Figure created with BioRender.com.

To determine the effects of chemogenetic activation or inhibition of KOR neurons in the CeA, we injected a Cre-inducible viral vector encoding either Gq-DREADD or Gi-DREADD into the right CeA (Figure 4A) and recorded from mCherry-expressing KOR neurons (see “Chemogenetic manipulations” and “Experimental protocol”) 4–5 weeks later (Figure 4B). We used electrophysiological recordings of CeA-KOR neurons expressing hM3Dq (Figure 4C) or hM4Di (Figure 4D) in brain slices from naïve KOR-Cre mice. DCZ (0.5 μM, 15 min) induced action potential firing (Figure 4(C3,C4)) and caused a significant depolarization (Figure 4(C5), *p* < 0.001, paired *t*-test, n = 6), indicating chemogenetic activation of CeA-KOR neurons. Increased action potential firing was found in 5 of 6 CeA-KOR neurons expressing hM3Dq (mCherry) (Figure 4(C6), *p* < 0.05, Chi-square test, n = 6). In CeA-KOR neurons expressing hM4Di, DCZ (0.5 μM, 15 min) caused a significant hyperpolarization (Figure 4(D3,D4), *p* < 0.01, paired *t*-test, n = 7) and decreased excitability measured as action potential firing in response to depolarizing current steps (Figure 4(D5,D6), *p* < 0.01, paired *t*-test, n = 7), indicating chemogenetic inhibition of CeA-KOR neurons. These results confirm that DCZ activated hM3Dq-expressing CeA-KOR neurons and inhibited hM4Di-expressing CeA-KOR neurons, thus supporting the chemogenetic strategy to modulate pain behaviors.

### 3.5. Effects of Chemogenetic Modulation of CeA-KOR Neurons on Downstream CeL Neurons

**Figure 5:**
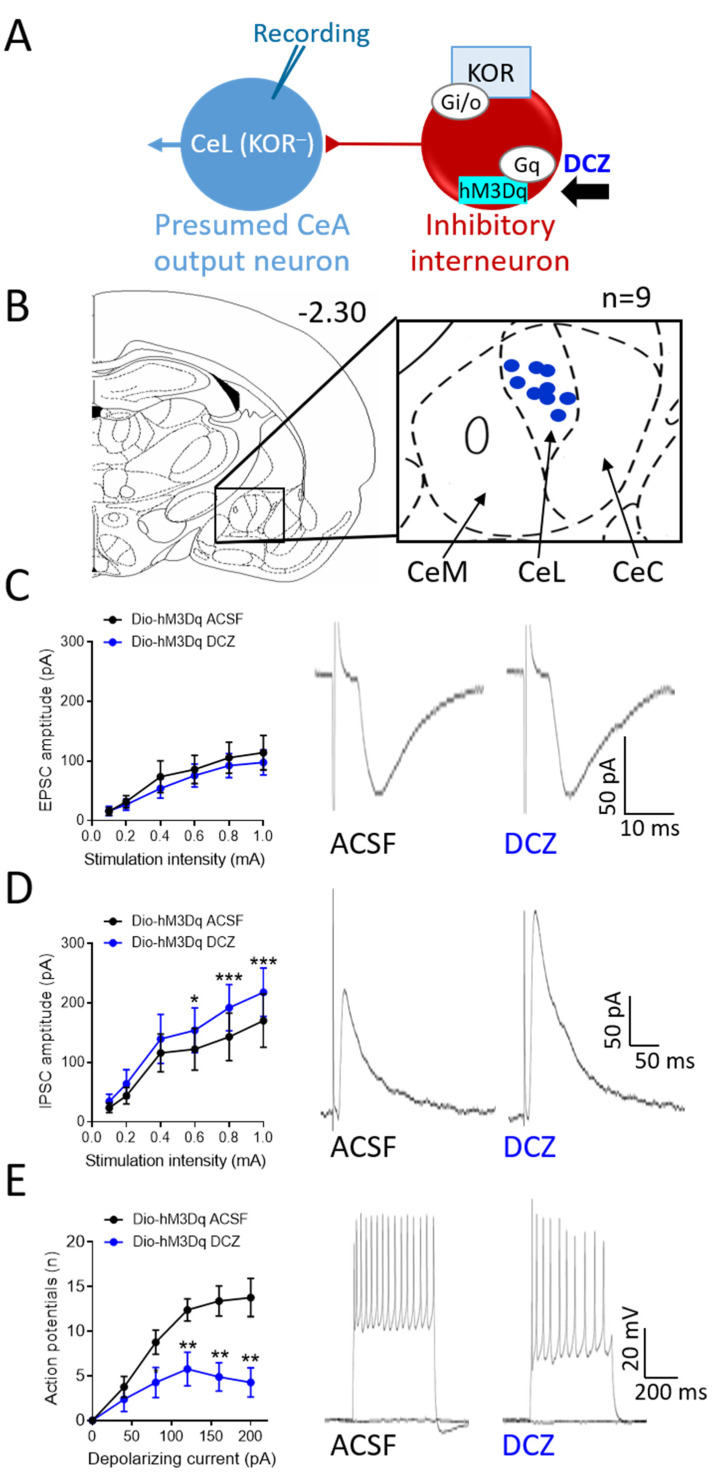
Effects of chemogenetic activation of CeA-KOR neurons on non-KOR neurons in the CeL in neuropathic KOR-Cre mice. (**A**) Diagram of hypothesis and experimental strategy. Whole-cell patch recording from non-KOR CeL neurons with chemogenetic activation (DCZ) of hM3Dq-expressing CeA-KOR neurons. (**B**) Coronal brain slice (2.30 mm caudal to bregma) shows recording sites of non-KOR neurons (n = 9) in the CeL in brain slices from neuropathic KOR-Cre mice (4 weeks post SNL induction). (**C**) Voltage-clamp recordings of evoked excitatory postsynaptic currents (EPSCs) in non-KOR CeL neurons. DCZ (0.5 µM, 15 min) had no significant effect (**left**: summary data, n = 9, two-way ANOVA with Bonferroni post hoc tests; **right**: individual traces). (**D**) Voltage-clamp recordings of evoked inhibitory postsynaptic currents (IPSCs). DCZ (0.5 µM, 15 min) increased IPSCs of non-KOR CeL neurons significantly (**left**: summary data, n = 8, * *p* < 0.05, *** *p* < 0.001, two-way ANOVA with Bonferroni post hoc tests; **right**: individual traces). (**E**) DCZ (0.5 µM, 15 min) decreased the frequency–current function of non-KOR CeL neurons significantly. Current–clamp recordings of action potentials generated by direct intracellular current injections (500 ms; 0 pA to 200 pA) from a holding potential of −60 mV before (ACSF) and during DCZ (0.5 µM, 15 min) application (**left**: summary data, n = 8, ** *p* < 0.01, two-way ANOVA with Bonferroni post hoc tests; **right**: individual traces).

**Figure 6:**
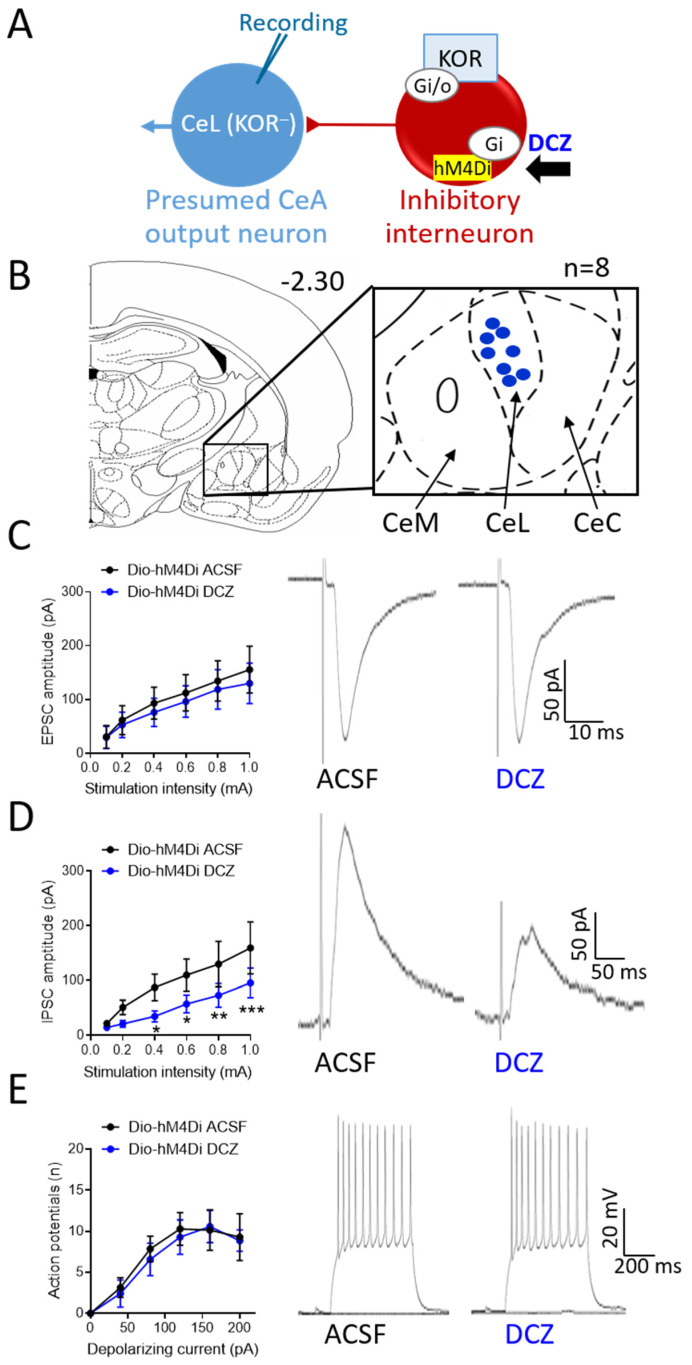
Effects of chemogenetic inhibition of CeA-KOR neurons on non-KOR neurons in the CeL in sham control KOR-Cre mice. (**A**) Diagram of hypothesis and experimental strategy. Whole-cell patch recording from non-KOR CeL neurons with chemogenetic inhibition (DCZ) of hM4Di-expressing CeA-KOR neurons in SNL KOR-Cre mice. (**B**) Coronal brain slice (2.30 mm caudal to bregma) shows recording sites of non-KOR neurons (n = 8) in the CeL in brain slices from sham KOR-Cre mice (4 weeks post-surgery). (**C**) Voltage–clamp recordings of evoked EPSCs. DCZ (0.5 µM) had no significant effects on EPSCs of non-KOR CeL neurons (left: summary data, n = 8, two-way ANOVA with Bonferroni post hoc tests; right: individual traces). (**D**) Voltage–clamp recordings of evoked IPSCs. DCZ (0.5 µM) decreased IPSCs in CeL neurons significantly (**left**: summary data, n = 8, * *p* < 0.05, ** *p* < 0.01, *** *p* < 0.001, two-way ANOVA with Bonferroni post hoc tests; **right**: individual traces). (**E**) DCZ (0.5 µM) had no significant effects on the frequency–current function of non-KOR CeL neurons. Current–clamp recordings of action potentials generated by direct intracellular current injections (500 ms; 0 pA to 200 pA) from a holding potential of −60 mV before (ACSF) and during DCZ (0.5 µM) application (**left**: summary data, n = 7, two-way ANOVA with Bonferroni post hoc tests; **right**: individual traces).

To test the hypothesis that KOR neurons in the CeA modulate amygdala activity by exerting an inhibitory tone on downstream CeA neurons (Figure 5A and Figure 6A), as proposed in our previous pharmacological studies [25,27], the effects of chemogenetic activation or inhibition of CeA-KOR neurons were tested on non-KOR neurons recorded in the CeL (Figure 5B and Figure 6B) using whole-cell patch–clamp.

Chemogenetic activation of hM3Dq-expressing CeA-KOR neurons with DCZ (0.5 µM, 15 min) in brain slices from SNL KOR-Cre mice (4 weeks post-induction) had no effect on evoked EPSCs of non-KOR neurons in the CeL (n = 9, Figure 5C left: summary data; right: individual traces), but significantly increased evoked IPSCs (n = 8, Figure 5D left: summary data, *p* < 0.05 and *p* < 0.001, two-way ANOVA with Bonferroni post hoc tests; Figure 5D right: individual traces; see “Brain Slice Electrophysiology”) compared to ACSF control. Neuronal excitability (frequency–current F-I function) measured in non-KOR neurons in the CeL (n = 8) as action potential firing generated by direct intracellular current injection (500 ms; 0 pA to 200 pA) was significantly decreased by DCZ (0.5 µM, 15 min) application compared to ACSF control (Figure 5E left: summary data, *p* < 0.01 two-way ANOVA with Bonferroni post hoc tests; Figure 5E right: individual traces).

Chemogenetic inhibition of hM4Di-expressing CeA-KOR neurons with DCZ (0.5 µM, 15 min) in brain slices from sham KOR-Cre mice (4 weeks post-surgery) had no effect on evoked EPSCs of non-KOR neurons in the CeL (n = 8, Figure 6C left: summary data; right: individual traces), but significantly decreased evoked IPSCs (n = 8, Figure 6D left: summary data, *p* < 0.05, *p* < 0.01, *p* < 0.001, two-way ANOVA with Bonferroni post hoc tests; Figure 6D right: individual traces; see “Brain Slice Electrophysiology”). DCZ (0.5 µM, 15 min) application had no significant effect on neuronal excitability (frequency–current function) of non-KOR neurons in the CeL (n = 7, Figure 6E left: summary data; Figure 6E right: individual traces). These data suggest that chemogenetic activation of CeA-KOR neurons can decrease excitability in sham mice via increased synaptic inhibition, whereas chemogenetic inhibition of CeA-KOR neurons decreases synaptic inhibition in SNL mice.

## 4. Discussion

The purpose of this study was to establish chemogenetics as a useful tool to investigate the specific role of amygdala KOR-expressing neurons and their downstream neuronal effects under chronic neuropathic pain and sham conditions. The amygdala is well established as a critical brain region in the emotional-affective aspects of pain and pain modulation [4,5,7,46], and evidence suggests that the KOR system may play an important role in stress responses and negative affective states via limbic brain regions such as the amygdala [17,47]. Therefore, KOR signaling within the amygdala, particularly in the CeA, may be linked to the averse-affective aspects of pain conditions. Previous work from our group demonstrated that KOR activation in naïve rats can increase amygdala output neuron activity and promote averse-affective pain-like behaviors [25], whereas KOR blockade in various pain conditions can increase inhibitory transmission onto amygdala output neurons and decrease pain-like behaviors [26,27,31,32,48].

However, prior studies explored the amygdala KOR system in pain via pharmacological manipulations using selective KOR agonists such as U-69,593 or selective KOR antagonists such as nor-binaltorphimine (nor-BNI). The actions of KOR-expressing neurons remained to be determined. Here, we characterized the functions of the amygdala (CeA) KOR system using a targeted chemogenetic approach to modulate KOR neurons. This technique allows for the selective and temporal activation or inactivation of KOR neurons with fewer off-target effects than traditional pharmacological methods [49,50]. In this study we measured the downstream neuronal and behavioral effects of KOR neuron inhibition in chronic neuropathic animals and in sham control animals and of KOR neuron activation in neuropathic animals. The hypothesis was that chemogenetic inhibition of KOR neurons would be similar to activation of Gi-coupled KOR, whereas chemogenetic activation could have effects that result, in part at least, from blockade of Gi-coupled KOR on these neurons, and we expected intrinsic KOR activation in the pain condition based on our previous data [26,27,32].

CeA output neurons are normally controlled via feedforward inhibition involving interneurons [6]. KOR-expressing CeA neurons may play a critical role in modulating neuronal activity. Opioid receptors are typically coupled to inhibitory G_i/o_-proteins and produce inhibitory effects on cellular signaling via reduced adenylate cyclase activity and a subsequent decrease in cAMP levels, leading to inhibition of calcium channels and opening of potassium channels [51]. As KOR-expressing neurons in the CeA are predominately GABAergic, activation of KOR by specific agonists results in a decreased firing rate and, as a consequence of this hypoactivity, relative disinhibition of efferent pathways, including the CRF and other amygdala output systems [25,27,52]. In contrast, removing inhibition of KOR neurons by pharmacological blockade of inhibitory KOR signaling or direct activation of KOR neurons enhances GABA release, which amplifies inhibitory signaling onto CeA output neurons and leads to a net decrease in CeA output signaling [6,16]. This dichotomy in the modulation of CeA output signaling, dependent on the mechanism of KOR neuron activation/inactivation, highlights the complexity of opioidergic control in this region. The consequences of direct activation or inhibition of KOR-expressing neurons rather than opioid receptors remain to be determined, and this knowledge gap was addressed in the present study using chemogenetic manipulations, as illustrated in Figure 5A and Figure 6A.

Here, the well-established SNL model of neuropathic pain was used to examine the effects of chemogenetic inhibition and activation of KOR-expressing CeA neurons on mechanical thresholds, emotional-affective responses, and anxiety-like behavior in KOR-Cre mice. We demonstrate that chemogenetic inhibition of CeA-KOR neurons can induce hypersensitivity and anxiety-like behaviors in sham mice but not in SNL mice (Figure 2D,F), suggesting that the neuropathic state may have altered the baseline functioning of the CeA to a degree that additional KOR neuron inhibition-induced CeA disinhibition does not produce a significant change in pain-related behavior. These results are consistent with the anxiogenic effects of intra-amygdala (basolateral) injection of dynorphin [53] and our previous report that a KOR agonist (U-69,593) administered into the CeA increased emotional-affective responses, anxiety-like behaviors, and avoidance behaviors as well as spinal nociceptive processing in naïve animals, likely via the disinhibition of CRF neurons [25,52]. Interestingly, pharmacological activation of KOR in the CeA in the same study had no significant effect on mechanosensitivity, possibly highlighting important differences between activation of KOR versus inhibition of KOR-expressing neurons.

Conversely, chemogenetic activation of KOR neurons in the CeA of SNL mice decreased sensory and emotional responses and anxiety-like behaviors, as demonstrated by increased withdrawal thresholds, fewer ultrasonic vocalizations in response to noxious stimuli, and increased time spent and entries into the light box, respectively (Figure 3D–G). This is consistent with prior studies showing that a KOR antagonist (nor-BNI) in the right CeA decreased CRF output signaling and emotional responses, anxiety-like behaviors, and hypersensitivity in a functional pain model [27] and relieved aversiveness, but not hypersensitivity, in a neuropathic pain model [26]. nor-BNI administered into the right (but not left) CeA prevented mechanical hypersensitivity in an injury-free model of functional cephalic pain [48], decreased facial mechanical hyperalgesia, but not anxiety-like behavior, in a model of trigeminal neuropathic pain [54], and restored descending pain modulation (diffuse noxious inhibitory control) that was impaired by stress [31] or neuropathic pain [32,55]. These results are consistent with evidence for increased dynorphin or dynorphin signaling in the amygdala in certain pain conditions [56,57]. The somewhat different findings with regard to sensory and anxiety-like behavioral modulation by KOR in the CeA could suggest limitations of pharmacological strategies. However, they are generally consistent with pain-facilitating versus inhibiting effects of direct chemogenetic inhibition versus activation of KOR neurons in the present study.

The chemogenetic manipulation of KOR neurons in the CeA reveals discrete changes in neuronal activity via temporal activation or inactivation that are otherwise difficult to discern using traditional pharmacological approaches, offering unique insights into pain-related signaling processes in this region. First, we validated the chemogenetic approach (Figure 4). Activation of Gq-DREADD (hM3Dq) expressing CeA-KOR neurons via DCZ resulted in significant membrane depolarization and increased neuronal firing in these neurons (Figure 4(C3,C4)). Conversely, inhibition of Gi-DREADD (hM4Di) expressing CeA-KOR neurons resulted in hyperpolarization and decreased action potential firing (Figure 4(D3–D6)). Future studies can build on these findings to achieve temporal control over specific neuronal activation or inhibition with the administration of the normally pharmacologically inert ligand DCZ [58,59], allowing for a more detailed investigation of the dynamic role of CeA-KOR neurons in pain-related processing. The chemogenetic approach is both repeatable and reversible [60,61], facilitating the longitudinal study of CeA-KOR activation/inactivation on chronic pain-related behaviors. Further investigation of this precise modulation of CeA-KOR neurons will provide a greater understanding of the dynamic role of the CeA-KOR system in pain-related signaling mechanisms.

The chemogenetic approach in the present study provides new insight into the neuronal actions of KOR in neuropathic pain. The specific synaptic and cellular mechanisms of KOR signaling within the CeA in pain-related conditions are not fully characterized. Our present findings agree with previously proposed KOR signaling mechanisms [25,26,27], as demonstrated by the chemogenetic activation (Figure 5) or inhibition (Figure 6) of CeA-KOR neurons. In SNL KOR-Cre mice, direct chemogenetic activation of hM3Dq-expressing CeA-KOR neurons with DCZ increased IPSCs (Figure 5D) but did not alter EPSCs (Figure 5C) in downstream CeL neurons, producing an increased inhibitory tone from KOR-expressing interneurons onto non-KOR CeL neurons to decrease their neuronal activity (Figure 5E). Conversely, in sham control KOR-Cre mice, direct chemogenetic inhibition of hM4Di-expressing CeA-KOR neurons did not affect EPSCs (Figure 6C) but decreased IPSCs (Figure 6D) of second-order CeL neurons, resulting in the suppression of inhibitory synaptic control within the CeA. Interestingly, chemogenetic inhibition did not affect the neuronal excitability of CeL neurons (Figure 6E), suggesting that, in the absence of a pain condition, the KOR-mediated inhibitory tone onto these downstream neurons is not sufficient to maintain baseline neuronal excitability. This may either be because normal synaptic inhibition is intact and sufficient or because signaling of other neuropeptides within the complex intra-CeA circuitry may counteract the effects of KOR neuron inhibition, as many have opposing effects on amygdala function [6]; the connectivity and receptor expression profiles of CeL neurons may dictate their responsiveness to KOR neuron activity. As a note of caution, while chemogenetics has revolutionized neuroscience research by providing new tools to manipulate the activity of specific cells and neural pathways, several limitations need to be considered. These include the diversity of viral vectors and expression variability, as well as properties of selective designer drugs, off-target effects, species differences, and others [28,62]. Some well-known issues concerning the use of CNO, including the need for high doses, the unstable crossing of the blood–brain barrier, low solubility in aqueous solution, and active metabolites, do not apply to this study because we used the highly selective and easily dissolvable DREADD actuator DCZ at a low dose. Control virus and saline control experiments were used to confirm the DREADD expression and DCZ effects.

It is important to note that while the cell type and projections of the CeL neurons in the current study were not identified, they may be CRF neurons based on their location and firing properties; however, the determination of both cell type and downstream targets should be further explored. Future studies could benefit from examining neuropeptide receptor expression on CeA output neurons and assessing their response to chemogenetic modulation to provide a greater understanding of the anatomical and functional roles of pain-related amygdala microcircuitry.

## 5. Conclusions

The present study elucidates the complex role of KOR signaling within the CeA and its downstream effects on CeL neuronal activity in pain modulation. Utilizing a chemogenetic approach, we demonstrate that direct activation of CeA-KOR neurons enhances inhibitory synaptic transmission and decreases neuronal excitability in neuropathic pain to inhibit pain-related behaviors. The chemogenetic inhibition of CeA-KOR neurons suppresses inhibitory transmission onto downstream CeA neurons under sham control conditions and induces pain-related behaviors. The findings highlight the critical role of the CeA-KOR system in neuropathic pain states and support the therapeutic potential of the targeted modulation of KOR neurons. Furthermore, the current study highlights the value of chemogenetic tools in dissecting the cellular and synaptic mechanisms of pain modulation, offering insights that may lead to the development of novel pain management strategies.

## Data Availability

The datasets generated during and/or analyzed during the current study are available from the corresponding author upon reasonable request.
